# Prayer and healing: A medical and scientific perspective on randomized controlled trials

**DOI:** 10.4103/0019-5545.58288

**Published:** 2009

**Authors:** Chittaranjan Andrade, Rajiv Radhakrishnan

**Affiliations:** Department of Psychopharmacology, National Institute of Mental Health and Neurosciences, Bangalore - 560 029, India; 1 Department of Psychopharmacology, National Institute of Mental Health and Neurosciences, Bangalore - 560 029, India

**Keywords:** Healing, miracles, prayer, randomized controlled trials, research design

## Abstract

Religious traditions across the world display beliefs in healing through prayer. The healing powers of prayer have been examined in triple-blind, randomized controlled trials. We illustrate randomized controlled trials on prayer and healing, with one study in each of different categories of outcome. We provide a critical analysis of the scientific and philosophical dimensions of such research. Prayer has been reported to improve outcomes in human as well as nonhuman species, to have no effect on outcomes, to worsen outcomes and to have retrospective healing effects. For a multitude of reasons, research on the healing effects of prayer is riddled with assumptions, challenges and contradictions that make the subject a scientific and religious minefield. We believe that the research has led nowhere, and that future research, if any, will forever be constrained by the scientific limitations that we outline.

*“More things are wrought by prayer*
*Than this world dreams of.”*
(Alfred, Lord Tennyson; from Morte d'Arthur)

*“Faith can move mountains.”*
(The Bible; paraphrased from Matthew 21:21)

## AUTHORS' PREFACE

This is a serious scientific article that examines conceptual and methodological issues underlying randomized controlled trials on prayer and healing. We do not intend to belittle any religion or the religious practices of those who pray, nor do we deny the medical and psychosocial benefits that have been identified to result from religious affiliations and practices.[1]

## INTRODUCTION

Religious practices have been associated with healing for millennia. People pray for good health and for relief from illness. Prayer may result in health and healing through one or more of several mechanisms. We briefly consider these mechanisms.

## MECHANISMS OF HEALING THROUGH PRAYER

### Prayer is a special form of meditation and may therefore convey all the health benefits that have been associated with meditation

Different types of meditation have been shown to result in psychological and biological changes that are actually or potentially associated with improved health. Meditation has been found to produce a clinically significant reduction in resting as well as ambulatory blood pressure,[2,3] to reduce heart rate,[4] to result in cardiorespiratory synchronization,[5] to alter levels of melatonin and serotonin,[6] to suppress corticostriatal glutamatergic neurotransmission,[7] to boost the immune response,[8] to decrease the levels of reactive oxygen species as measured by ultraweak photon emission,[9] to reduce stress and promote positive mood states,[10] to reduce anxiety and pain and enhance self-esteem[11] and to have a favorable influence on overall and spiritual quality of life in late-stage disease.[12] Interestingly, spiritual meditation has been found to be superior to secular meditation and relaxation in terms of decrease in anxiety and improvement in positive mood, spiritual health, spiritual experiences and tolerance to pain.[13]

### Prayer may be supported by varying degrees of faith and may therefore be associated with all the benefits that have been associated with the placebo response

Clinically significant treatment gains have been observed with placebo in numerous disorders, including anxiety, depression, schizophrenia, obsessive-compulsive disorder, tardive dyskinesia, ischemic heart disease, cardiac failure, Parkinson's disease and even cancer, among a host of other conditions.[14–20] Relevant to the context of prayer and healing, the placebo response is influenced by personality traits and behaviors such as optimism,[21,22] response expectancy,[23] motivational concordance (i.e., the degree to which the behavioral rituals of the therapy are congruent with the motivational system of the subject)[24] and degree of engagement with a ritual.[25]

### Prayer may be associated with improvements that result from spontaneous remission, regression to the mean, nonspecific psychosocial support, the Hawthorne effect and the Rosenthal effect

Spontaneous remission is well known to occur in conditions that range from medical disorders (e.g., coryza and pharyngitis) to psychiatric states (e.g., depression and mania). Regression to the mean describes improvement that occurs as a result of random fluctuation in the severity of illness; in clinical trials, because patients are usually preselected for greater severity of illness, such fluctuations usually occur in only one direction (i.e., toward improvement).[26] Nonspecific emotional support provides psychological benefits through interpersonal contact, such as during diagnostic and rating exercises. Nonspecific support can reduce anxiety, depression, pain and similar constructs.

Spontaneous remission and regression to the mean may occur coincidental to prayer. Nonspecific psychosocial support related to prayer may arise in group prayer settings. Improvements in all these contexts are true improvements. In contrast, in randomized controlled studies on the efficacy of prayer as a treatment, rated improvements that are not true improvements may also occur; explanations for such improvement include the Hawthorne effect and the Rosenthal effect. The Hawthorne effect refers to change that occurs as a result of the act of observation or measurement,[27,28] whereas the Rosenthal effect refers to change resulting from observer or rater expectancy.[29] With regard to the former, the comforting environment of the study setting or the conscious or unconscious wish of the patient to please may result in the report of less symptoms than actually exist. With regard to the latter, the tendency of the rater to expect symptom attenuation across time may result in the attachment of lower significance to reported symptoms.

### Prayer may result in benefits that are due to divine intervention

Although the very consideration of such a possibility may appear scientifically bizarre, it cannot be denied that, across the planet, people pray for health and for relief of symptoms in times of sickness. Healing through prayer, healing through religious rituals, healing at places of pilgrimage and healing through related forms of intervention are well-established traditions in many religions.

## DIVINE INTERVENTION AS A MECHANISM OF HEALING THROUGH PRAYER

Meditation, the placebo response, regression to the mean, the natural course of various illnesses, nonspecific emotional support, the Hawthorne effect and the Rosenthal effect have all been studied. What about divine intervention as a mechanism of recovery of health through prayer? This has also been seriously investigated.

Astin *et al.*[30] conducted a systematic review of the literature on the efficacy of any form of distant healing as a treatment for any medical condition. A total of 23 trials involving 2,774 patients met the inclusion criteria and were subjected to analysis. Of these studies, 13 (57%) yielded statistically significant treatment effects favoring distant healing, nine showed no superiority of distant healing over control interventions and one showed a negative effect for distant healing. The methodological limitations of many of the studies, however, made it difficult to draw definitive conclusions about the efficacy of distant healing. Of note, Astin *et al.*[30] defined distant healing to include spiritual healing, prayer, and any form of healing from a distance, effected as a conscious act that seeks to benefit another person. Therapeutic touch and Reiki were both included in the definition; as both of these may elicit an expectancy response,[31] it becomes even harder to draw definitive conclusions about the literature that Astin *et al.*[30] examined.

In another systematic review, Crawford *et al.*[31] examined the quality of studies of hands-on healing and distance healing that were published between 1955 and 2001. There were 90 identified studies of which 45 had been conducted in clinical settings and 45 in laboratory settings. Crawford *et al.*[31] reported that 71% of the clinical studies and 62% of the laboratory studies reported positive outcomes; and that the overall internal validity for the studies on distance healing was 75% for the clinical investigations and 81% for the laboratory investigations. Major methodological problems of the identified studies were an inadequacy of blinding, dropped data in laboratory studies, unreliability of outcome measures, infrequent use of power estimations and confidence intervals, and lack of independent replication.

In the present article, we present a purposive, qualitative review of the scientific literature on possible paranormal healing through prayer. We then critically evaluate the scientific and religious implications of such research.

## MATERIALS AND METHODS

The currently accepted gold standard for the investigation of the efficacy of medical interventions is the double-blind, randomized controlled trial. Most recent studies on prayer and healing have adopted this design. In such studies, commonly, a group of intercessors prays for the health of patients who are randomized to the intervention group. These patients do not know that they are being prayed for, and the persons who are praying do not come in contact with the patients for whom they pray. Medical outcomes in these patients are compared with outcomes in patients randomized to the control group who are not prayed for. Finally and importantly, the medical treatment team is also blind to the prayer group status of individual patients. Thus, these studies are triple-blind.

In this purposive review, we illustrate the nature of the research in the field by presenting one human and one nonhuman study on improved outcomes associated with prayer, one study showing no difference between prayer and control conditions, one study showing worse outcomes with prayer and one study suggesting that prayer may have a retrospective healing effect. We then provide a detailed, critical evaluation of the scientific and theological implications of such research.

## RESULTS

### Improved outcomes associated with prayer

Cha *et al.*[32] studied 219 consecutive infertile women, aged 26-46 years, who were treated with *in vitro* fertilization embryo transfer in Seoul, South Korea. These women were randomized into distant prayer and control groups. Prayer was conducted by prayer groups in the USA, Canada and Australia. The patients and their providers were not informed about the intervention. The investigators, and even the statisticians, did not know the group allocations until all the data had been collected. Thus, the study was randomized, triple-blind, controlled and prospective in design.

Cha *et al.*[32] found that the women who had been prayed for had nearly twice as high a pregnancy rate as those who had not been prayed for (50 vs. 26%; *P* <0.005). Furthermore, the women who had been prayed for showed a higher implantation rate than those who had not been prayed for (16.3 vs. 8%; *P* <0.001). Finally, the benefits of prayer were independent of clinical or laboratory providers and clinical variables. Thus, this study showed that distant prayer facilitates implantation and pregnancy.

Lesniak[33] described a study on the effect of intercessory prayer on wound healing in a nonhuman primate species. The sample comprised 22 bush babies (*Otolemur garnettii*) with wounds resulting from chronic self-injurious behavior. These animals were randomized into prayer and control groups that were similar at baseline. Prayer was conducted for 4 weeks. Both groups of bush babies additionally received L-tryptophan. Lesniak[33] found that the prayer group animals had a greater reduction in wound size and a greater improvement in hematological parameters than the control animals. This study is important because it was conducted in a nonhuman species; therefore, the likelihood of a placebo effect was removed.

### Absence of benefits with prayer

Aviles *et al.*[34] examined cardiovascular outcomes related to prayer. In this study, 799 coronary care unit patients at discharge were randomized to intercessory prayer or no prayer conditions. Prayer was conducted by five persons per patient at least once a week for 26 weeks.

Patients were considered to belong to a high-risk group if they were 70 years old or older or if they had any of the following: diabetes mellitus, previous myocardial infarction, cerebrovascular disease or peripheral vascular disease. The primary endpoint of the study was any of the following: death, cardiac arrest, rehospitalization for cardiovascular disease, coronary revascularization or an emergency department visit for cardiovascular disease.

By the end of 26 weeks, a primary endpoint had occurred in 25.6% of patients in the prayer group and in 29.3% of patients in the control group. The difference was not statistically significant. The results remained nonsignificant when data were analyzed separately for high- and low-risk patients. Thus, this study showed that, as delivered in this study, intercessory prayer did not influence the 26-week outcome after discharge from a coronary care unit.

Other recent randomized controlled trials have also reported negative results. For example, Krucoff *et al.*[35] reported no benefits with off-site prayer in patients (*n* = 748) undergoing percutaneous coronary interventions and Astin *et al.*[36] found that neither remote prayer delivered by professional healers nor remote prayer delivered by nurses with no training or experience in distance healing resulted in benefits to patients (*n* = 156) with acquired immunodeficiency syndrome-defining opportunistic infections.

### Worse outcomes associated with prayer

Benson *et al.*[37] described a triple-blind, randomized controlled study that examined whether remote intercessory prayer influenced recovery after coronary artery bypass graft surgery and whether the certainty of being prayed for was associated with better outcomes. The sample comprised 1,802 patients in six hospitals in the USA. These patients were randomized into three groups: 604 were prayed for after being informed that they may or may not be prayed for, 597 were not prayed for after similarly being informed that they may or may not be prayed for and 601 were prayed for after being informed they would definitely be prayed for.

Prayer commenced one day before the surgery and continued for 14 days. Three mainstream religious sites prayed daily for patients assigned to receive prayer. Assessment of outcomes was made by nurses who were blind to the group assignments. The primary outcome was the presence of any complication within 30 days of surgery. Secondary outcomes were any major event, including death. The study sought to examine the efficacy of intercessory prayer and not to test the presence of God. The design was described by Dusek *et al.*[38]

In the two groups that did not know for certain whether or not they were being prayed for, complications occurred in 52% of patients who received intercessory prayer and in 51% of those who did not. In contrast, complications occurred in a significantly larger proportion of patients (59%) who knew for certain that they were being prayed for. Major events and 30-day mortality rates, however, were similar across the three groups.

This study therefore showed that remote intercessory prayer did not improve outcomes after coronary artery bypass graft surgery. In fact, the knowledge of being prayed for was associated with a slightly but significantly higher rate of postsurgical complications.

### Retrospective benefits with prayer

Leibovici[39] reported the results of an unusual study that was conducted in Israel. The sample comprised 3,393 in patients diagnosed with a bloodstream infection between 1990 and 1996. Bloodstream infection was defined as a positive blood culture in the presence of sepsis.

These patients were randomized into prayer (*n* = 1,691) and control (*n* = 1,702) groups in July, 2000. A list of the first names of the patients in the prayer group was given to a person (details not specified) who said a short prayer (details again not specified) for the wellbeing and full recovery of the group as a whole. This prayer was said about 4-10 years or longer after the index admission. There was no sham intervention. Thus, this study sought to determine whether prayer has a retrospective healing effect.

The patients in the prayer and control groups were similar on important sociodemographic and clinical variables. Whereas the mortality rate did not differ significantly between the prayer and the control groups (28.1 vs. 30.2%, respectively), the length of stay in the hospital and the duration of fever were both significantly shorter in the prayer group than in the control group (*P* = 0.01 and 0.04, respectively).

Some points about this study are worth noting. The differences between groups, although significantly favoring patients for whom prayer was offered, were very small; the medians of the two groups differed by a small margin. Thus, the significance of the findings depended heavily upon the outliers who skewed the sample. Next, no attempt was made to compare for unusual biases, such as day of admission and discharge. It is conceivable, for example, that patients admitted toward the end of the week may have been investigated and treated more slowly and those due for discharge toward the end of the week may have been retained until the start of the next week.

Importantly, considering the number of patients in each group, there must surely have been much overlap in first names. Did Leibovici consider the possibility that the prayers, then, could benefit patients in both groups to the extent of overlap? Finally, in a lighter vein, would the findings have changed had the author, in the best spirits of ethical research, offered the experimental intervention (prayer) for the control group at the conclusion of the study? More seriously, because the data were retrospective, it should have been possible for the study to have been repeated several times, with fresh randomization each time. Would the results, then, have remained unchanged? These and other issues were raised in the journal correspondence published on the Leibovici[39] article.

## DISCUSSION

In the broadest sense, prayer describes thoughts, words or deeds that address or petition a divine entity or force. Chibnall *et al.*[40] and Sloan and Ramakrishnan[41] critically discussed the growing body of research on the healing effects of distant intercessory prayer. We expand on certain of their views and of the views expressed in the journal correspondence that followed their article, and we add our critical perspectives in the discussion that follows. Some technical notes that do not flow with the text are provided in the Appendix.

By invoking prayer, researchers invite troublesome questions about the importance of several theosophical matters:

1. Do the quantitative aspects of prayer influence outcomes? Quantity refers to the number of prayers, the frequency of the prayers and the duration of the prayers.
2. Do the qualitative aspects of prayer influence outcomes? Quality refers to the category to which the prayer belongs in the religion of the person who is praying; the fervency with which the petition is expressed; whether the prayer is expressed in thoughts, speech or song; the addition of vows and sacrifices, etc.
3. Does the practical content of the prayer or the actual petition matter? That is, are some petitions more or less likely to receive a favorable response, depending on how reasonable they are?
4. Are outcomes more likely to be favorable if the persons praying have greater belief that the outcome will be favorable, or greater faith or conviction in the deity at whom the prayer is directed?
5. Are outcomes more likely to be favorable if a larger number of people pray or if a team approach is adopted as opposed to an individual approach?
6. Might outcomes depend on the personal characteristics of the persons who pray; that is, their age, sex, income, religious denomination, position in the religious hierarchy, experience with and skills at praying and so on?
7. Might outcomes depend on the moral and social characteristics of the persons who pray; that is, their integrity, kindness, altruism, willingness to forgive, generosity, religiosity and so on?
8. Might outcomes depend on the personal, moral and social characteristics of the persons in whose favor the prayer is offered, or of the general worthiness of the cause?
9. Would the outcomes depend on the entity at whom the prayers are directed?
10. What is the nature and magnitude of response that would be considered as a favorable outcome?

These “pharmacokinetic and pharmacodynamic” descriptors of prayer are all important issues to judge from the manner in which persons pray, or if persons with strong religious affiliations are to be believed. Therefore, all of the above would need to be considered as independent or confounding variables in any scientific study on the efficacy of intercessory prayer. Curiously, no study has so far addressed these issues. And, for several reasons, such issues are disturbing because they reduce the concept of God to that of a human being with weaknesses and vanities, thereby exposing theological inconsistencies and attacking the very roots of theology and natural justice. We present some of the unsettling questions that arise in these contexts; the questions are unsettling because they invite comparison with human parallels that devalue the concept of God, something that those who pray surely would not have considered.

1. If the number, duration and frequency of prayer are important or if the number of persons praying is important, does God, like a businessman, market boons based on the currency value of the prayers? Or, will God pay attention only if those who pray are sufficiently bothersome?
2. If the type of prayer is important, is God a bureaucrat who is more likely to consider petitions that appear in the prescribed forms?
3. If the addition of vows and sacrifices is important, is God somebody who can be flattered or bribed into granting a boon?
4. If the level of fervency or intensity is important, does God distinguish between “please”, “pretty please” and “pretty please with ribbons on it”?
5. If the practical content of and petitions in the prayer are important, how does God make decisions about what is and what is not a reasonable request?
6. If the faith or conviction of the persons who pray is important, does God value the beliefs of the petitioners more than the merits of the petitions?
7. If the personal characteristics and qualities of the persons who pray (or the persons who are being prayed for) are important, are some people more equal before God than other people? Religions portray God as being compassionate; what sort of compassion is displayed by the selective favoring of an experimental over a control group?
8. If the entity to which the prayer is directed is important, do different Gods have different portfolios? Are some Gods more approachable? Do some Gods ignore some prayers? If the religious affiliation of the person who prays is important, what becomes of the other religions of the world and those who follow such religions; will their prayers remain unanswered?
9. If the magnitude of response to the petitions is total, then all prayers should result in miraculous or near-miraculous benefits. This, clearly, almost never happens. Thus, does God work on percentages; that is, if the petition is for an elephant, does he sanction a mouse? Or, are his responses only subtle ones? If so, how does he choose on the outcome measure to improve?

These questions are unsettling to those who pray because of their theological implications, but they are also unsettling to scientists because they challenge the design, analysis and interpretation of randomized controlled trials of the efficacy of intercessory prayer. Consider the following:

1. It could be difficult, if not impossible, to measure all the independent and confounding variables that are important in such research. For example, how might one measure faith, fervency, reasonableness, worthiness, religiosity, morality and other abstract constructs?
2. How might one define what is an acceptable response to prayer? Healing can be partial or complete. It can be psychological or physical. It can be abstract or concrete. Confounding the picture, statistically significant improvement can be identified only if the same outcome measure is improved in a sufficiently large number of experimental relative to control patients, but why should God decide to select any one outcome measure over the rest? And if different outcome measures improve in different experimental patients in response to prayer, there is no way in which the improvement can be statistically detected.
3. As atheists, in general, form a minority in most populations, in any randomized controlled trial of intercessory prayer, there is likely to be a number of persons (friends, relatives and the patients themselves) praying for members of both experimental and control groups, unknown to the researchers. If prayer works, this unmeasured source of healing could diminish intergroup differences in outcomes.
4. As inferential statistical tests will be applied to the data generated by randomized controlled trials of intercessory prayer, is it valid to assume that acts of God conform to normal, t or other statistical distributions? Or that God responds mechanistically to prayer, in a manner that follows laws of probability? In this context, miraculous healings are considered to be outside the provisions of nature, and so divine intervention could actually be expected to violate probability.
5. Alternately, if prayer is a nonlinear variable, the merits and demerits of which are decided upon by God, then one prayer made by a control patient or relative can statistically offset a multitude of intercessory prayers offered on behalf of the experimental patients. In fact, if divine intervention is selective or arbitrary in response to petitions, the entire basis of randomized controlled design and inferential statistical analysis becomes invalid.

From a scientific perspective, if prayer is indeed considered to work, thought should also be given to the possibility that it may not require a deity. It may, instead, invoke some hitherto unidentified mental energy that has healing power. If so, might prayer be more effective if those who pray are in closer proximity to those who are being prayed for? Might the direction in which persons face (while praying) matter? Might the assistance of the physical sciences be required to identify the nature of the biological energies at work?

It should be noted that the distant healing, intercessory prayer studies specifically test the intervention of a divine entity. This is because the intercessors are usually blind to the identities of the patients for whom they pray, or (at least) because the intercessors do not have any contact with these patients. Therefore, it is left to a sentient being to miraculously divine the intent of the prayers and apply the intercession to the correct target.

Of note, distant healing, intercessory prayer studies address soft diagnoses with soft outcomes. No study, for example, has examined whether prayer can result in the disappearance of medically proven tumors and metastases, reversal of traumatic paraplegia or revival from a state of brain death. It would seem that the results of such studies could be more convincing than the results of studies on wound healing or successful pregnancy. Could it be that those who pray believe that God has or sets limitations?

We close our critique with two final questions:

1. If research on intercessory prayer is positive, does it suggest to us ways and means by which we can manipulate God or make his behavior statistically predictable?
2. Why would any divine entity be willing to submit to experiments that attempt to validate his existence and constrain his responses?

In this context, we must keep in mind that religion is based on faith and not on proof. This implies that, if God exists, he is indifferent to humanity or has chosen to obscure his presence. Either way, he would be unlikely to cooperate in scientific studies that seek to test his existence.

Where does this leave us? God may indeed exist and prayer may indeed heal; however, it appears that, for important theological and scientific reasons, randomized controlled studies cannot be applied to the study of the efficacy of prayer in healing. In fact, no form of scientific enquiry presently available can suitably address the subject. Therefore, the continuance of such research may result in the conducted studies finding place among other seemingly impeccable studies with seemingly absurd claims (Renckens *et al.*[42] 2002). Whereas we have attempted to be scientifically and politically correct in our critique, other authors, such as Dawkins,[43] have been humorous, nay even scathing, in their criticism.

The aim of science is not to open a door to infinite wisdom but to set a limit to infinite error (attr., Galileo[44]).

## Appendix

**Table:**

Technical notes about the conduct, description and analysis of randomized controlled trials on remote intercessory prayer and healing
The CONSORT statement may not be adequate for reporting trials that use nontraditional interventions such as prayer. In this context, the Standards for Reporting Interventions in Controlled Trials of Acupuncture (STRICTA) group has developed an extension of the reporting requirements relevant to clinical trials of acupuncture.[45] Scientists involved in prayer research, however, have no similar guidelines to follow. Dusek *et al.*,[46] describe consensus recommendations that require trials to provide greater details about the exact nature and content of interventions, details regarding patient personality dispositions, details regarding the “dose” of prayer, patient awareness of and blinding to the intervention and prayer logs that document the validity of the intervention, among other issues. A Bayesian approach to the analysis of research of this nature may carry advantages over the conventional approaches.[47,48]
